# Acidic extracellular pH promotes epithelial mesenchymal transition in Lewis lung carcinoma model

**DOI:** 10.1186/s12935-014-0129-1

**Published:** 2014-11-30

**Authors:** Atsuko Suzuki, Toyonobu Maeda, Yuh Baba, Kazuhiro Shimamura, Yasumasa Kato

**Affiliations:** Department of Oral Function and Molecular Biology, Ohu University School of Dentistry, Koriyama, Japan; Department of General Clinical Medicine, Ohu University School of Dentistry, Koriyama, Japan; Department of Oral Growth and Development, Ohu University School of Dentistry, Koriyama, Japan; Department of Oral Physiology and Biochemistry, Ohu University Graduate School of Dentistry, Koriyama, Japan

**Keywords:** Acidic extracellular pH, Invasion, EMT, Lewis lung carcinoma, MMP-9

## Abstract

**Background:**

Epithelial mesenchymal transition (EMT) is thought to be an essential feature of malignant tumor cells when they spread into the stroma. Despite the extracellular acidity of tumor tissues, the effect of acidic extracellular pH (pH_*e*_) on EMT in carcinoma models, including the Lewis lung carcinoma (LLC) model, remains unclear.

**Methods:**

High and low metastatic LLC variants were generated by repeated tail vein injection of metastatic cells. DMEM/F12 medium, which has been supplemented with 15 mM HEPES, 4 mM phosphoric acid, and 1 g/L NaHCO_3_ and adjusted to the desire pH with HCl or NaOH, was used for cell culture. EMT marker gene expression was determined by quantitative reverse transcription-polymerase chain reaction. Migration and invasion activities were analyzed by wound healing assay and the Boyden chamber assay through Matrigel®, respectively.

**Results:**

Low metastatic variant LLCm1 cells showed a cobble-stone like morphology at pH_*e*_ 7.4. At pH_*e*_ 6.8, however, their morphology became fibroblastic, similar in shape to high metastatic variant LLCm4 cells. Steady state levels of matrix metalloproteinase-9 (*Mmp9*) mRNA were induced by acidic pH_*e*_, maximizing at pH 6.8, with the levels of *Mmp9* mRNA higher in LLCm4 than in LLCm1 cells. Both variants showed decreased levels of E-cadherin and increased levels of vimentin at pH_*e*_ 6.8. Acidic pH_*e*_ also induced expression of mRNAs encoding the E-cadherin repressors, *Zeb2*, *Twist1* and *Twist2*, as well as enhancing cell motility and *in vitro* invasion through Matrigel®.

**Conclusions:**

Acidic pH_*e*_ can induce EMT in some types of carcinoma.

## Background

Cancer metastasis involves complex steps. The invasion of cancer cells into surrounding tissues is accompanied by a change in properties from epithelial to mesenchymal, *i.e*., epithelial mesenchymal transition (EMT). EMT can be induced by cytokines and growth factors, such as epidermal growth factor (EGF), basic fibroblast growth factor (bFGF/FGF2), and transforming growth factor-β (TGF-β), either alone or in combinations. EMT is also accompanied by up-regulation of the expression of vimentin, N-cadherin, and E-cadherin repressors such as Snail, Slug, Twist1, Twist2, Zeb1, and Zeb2 (see [1] for review). Down-regulation of E-cadherin causes dysfunction at adherence junctions, leading to detachment of cancer cells from their primary site [2].

Acidic extracellular pH (pH_*e*_) is an important feature of solid tumors [3] that induces tumor metastasis [4]. Indeed, acidic pH_*e*_ is an important microenvironmental factor in metastasis induction [5]. Using mouse metastatic B16 melanoma cells, we found that acidic pH_*e*_ induces cellular expression of matrix metalloproteinase-9 (MMP-9) and induces morphological changes to a fibroblastic phenotype [6]. In addition, acidic pH_*e*_ was found to induce EMT-like changes in human melanoma cells [7]. Melanomas are tumors that are transformed from melanocytes derived from the neural crest, but do not show typical properties of EMT [8]. These results suggested that acidic pH_*e*_ may act as a microenvironment inducing EMT in carcinoma models. Here, we tested this hypothesis using two variants of Lewis lung carcinoma (LLC) with different metastatic activities.

## Results

### Establishment of LLC variants with different metastatic abilities

**Figure 1:**
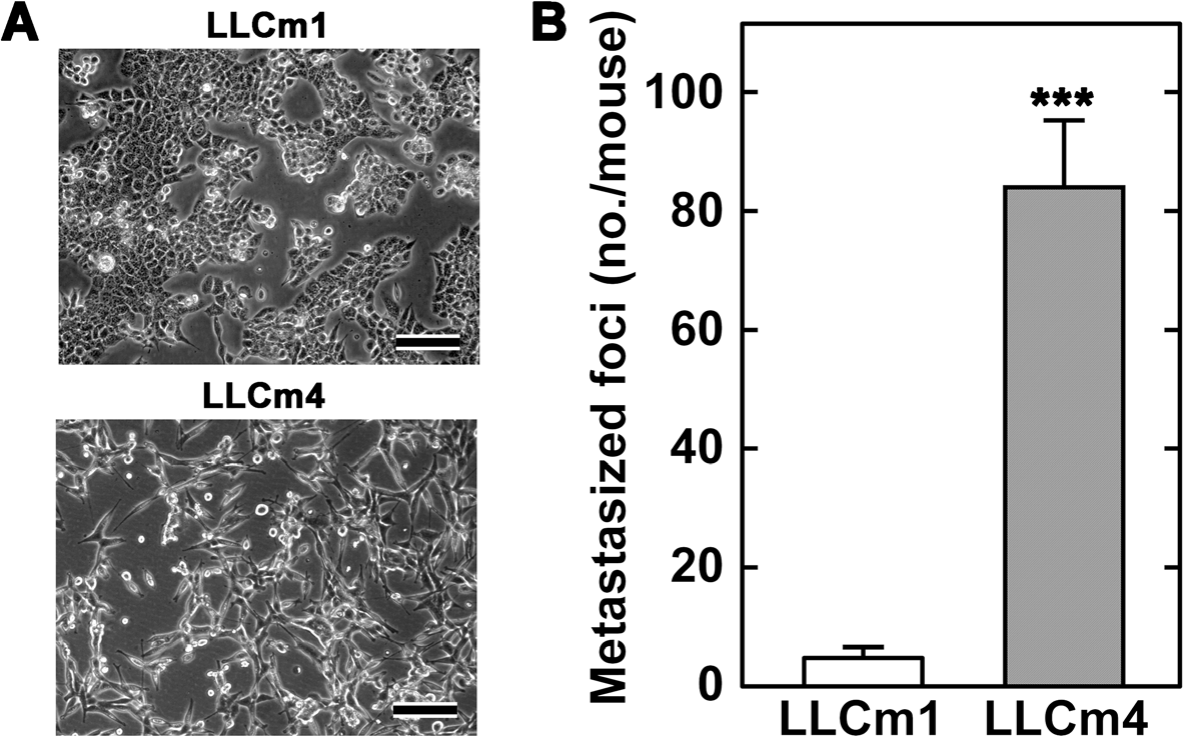
**Morphology and metastatic abilities of LLC variants. A**. Photographs taken under an inverted phase contrast microscope of logarithmic phase cells grown in 10% FBS-containing medium. Bar, 100 μm. **B**. Cells were injected into the tail veins of syngeneic mice. Three weeks later, foci that had metastasized to the lungs were counted. Representative results were shown from two independent experiments. ***P<0.001.

To compare the effect of acidic pH_*e*_ on metastatic potential, we established two LLC variants (LLCm1 and LLCm4) by repeating cycles of the experimental pulmonary metastases. LLCm1 cells had a cobble-stone like morphology and were tightly attached to each other, whereas LLCm4 cells had a spindle shaped morphology and were scattered (Figure 1A). The metastatic potential of LLCm4 cells was 9 times higher than that of LLCm1 cells (Figure 1B).

### Acidic pH_*e*_ induces MMP-9 production

**Figure 2:**
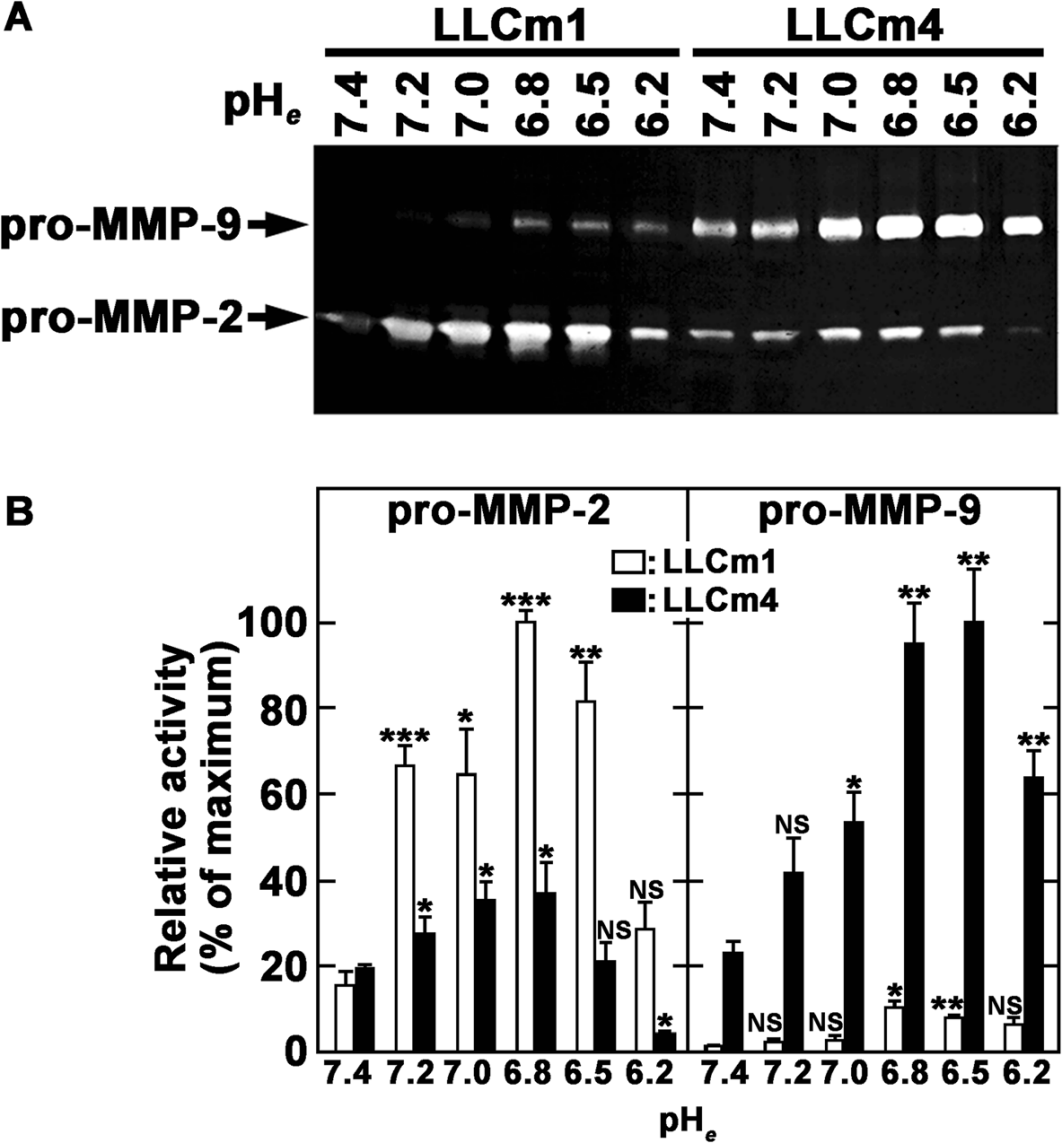
**Acidic pH**_***e***_ **induces MMP-9 production.** Sub-confluent cells were cultured overnight in serum-free medium at pH 7.4 and stimulated with serum-free medium at the indicated pH. After 24 h, the conditioned medium was collected, concentrated, and analyzed by gelatin zymography. **A**. A clear zone indicates gelatinolytic activity. **B**. Intensity of zymogram was quantified by Scion Image (Scion corp., Frederick, MD, USA). Representative results were shown from three independent experiments. *P < 0.01; **P < 0.01; ***P < 0.001; NS, not significant.

As shown previously, MMP-9 is an acidic pH_*e*_-signal target gene, with the acid-induced level of MMP-9, but not MMP-2, expression positively correlated with the metastatic potential of mouse B16 melanoma variants [6]. We therefore assessed whether acidic pH_*e*_ induces MMP-9 production, and whether its level correlated with the metastatic potential of the two LLC variants. MMP-9 production by both variants was clearly pH_*e*_ dependent (Figure 2), with maximal production at pH_*e*_ 6.8. The acid pH_*e*_ stimulated expression of MMP-9 was much higher by LLCm4 than by LLCm1 cells, while LLCm4 cells produced constitutively higher level of MMP-9 as the basal level at pH_*e*_ 7.4. Although MMP-2 level was also slightly induced by acidic pH_*e*_, the highly metastatic LLCm4 cells produced much less MMP-2 than LLCm1 cells. This finding was in accordance with the mouse B16 melanoma model, in that MMP-2 levels are high in parental B16 cells but negligible in highly metastatic B16-F10 and B16-BL6 cells [6].

**Figure 3:**
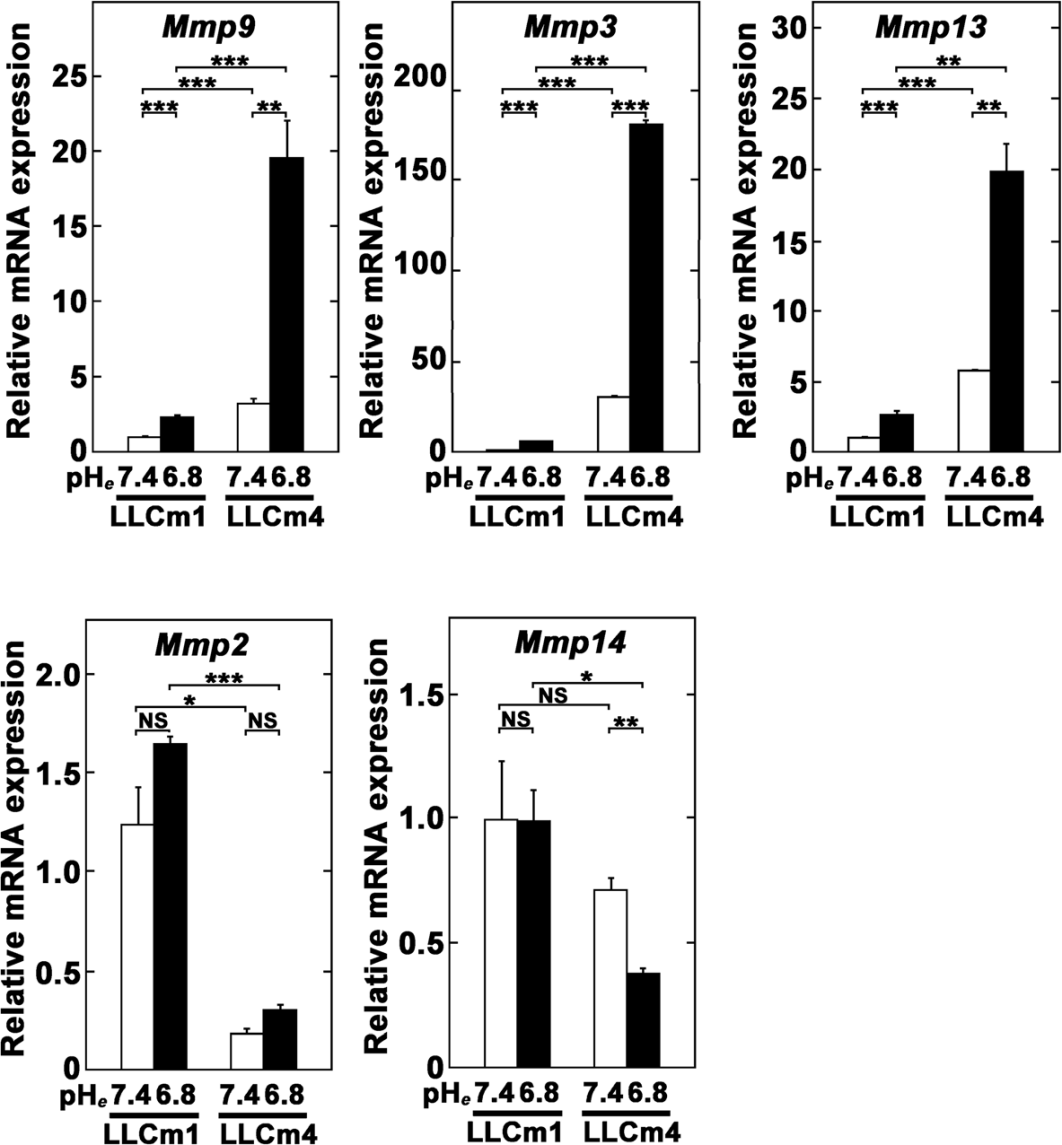
**Acidic pH**_***e***_ **induces not only** ***Mmp9*** **but also** ***Mmp3*** **and** ***Mmp13*** **mRNA expressions but not obvious** ***Mmp2*** **and** ***Mmp14*** **mRNA expressions.** Sub-confluent cell cultures were pretreated overnight with serum-free medium at pH 7.4 and stimulated by serum-free media at pH 6.8 or 7.4. After 24 h, total RNA was extracted, reverse-transcribed, and amplified by qPCR with specific primer sets. Data are shown as relative expression compared with LLCm1 cells cultured at pH 7.4. Representative results were shown from three or more independent experiments. **P < 0.01; ***P < 0.001; NS, not significant.

MMP-9 induction by acidic pH_*e*_ was also confirmed by reverse transcription-quantitative polymerase chain reaction (RT-qPCR). Accordance with zymographic analysis (Figure 2), the basal level of *Mmp9* mRNA at pH_*e*_ 7.4 was higher in LLCm4 cells than LLCm1 cells. Acid pH_*e*_ enhanced the steady state levels of *Mmp9* mRNA 2-fold in LLCm1 cells and 6-fold in LLCm4 cells (Figure 3). In addition to *Mmp9*, *Mmp3*, and *Mmp13* mRNA expressions were also stimulated by acidic pH_*e*_. Although induction of MMP-2 secretion was observed (see Figure 2), increase in *Mmp2* mRNA expression was not statistically significant, suggesting a possibility that acidic pH_*e*_ affects the efficiency of protein translation. *Mmp14* mRNA, whose protein is critical for MMP-2 activation, was higher in LLCm1 cells than LLCm4 cells and was not affected by acidic pH_*e*_.

### Acidic pH_*e*_ induces morphological changes to a fibroblastic morphology

**Figure 4:**
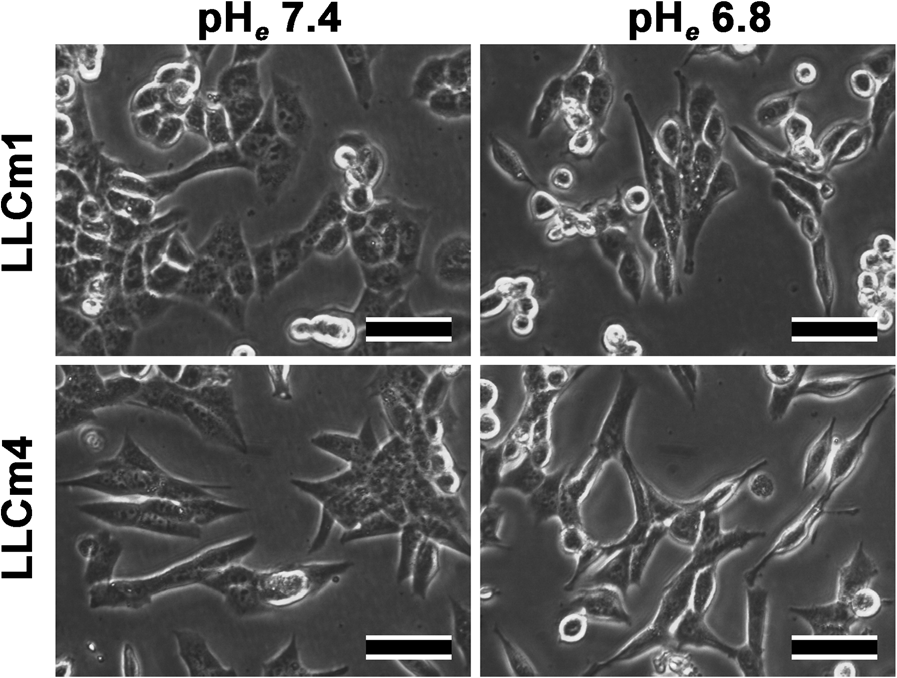
**Morphological changes of LLC variants in acidic medium.** Cultures were pretreated overnight with serum-free DMEM/12 at pH 7.4 and incubated in serum-free medium at pH 6.8 or 7.4 for 24 h. Photographs were taken under an inverted phase contrast microscope. Representative results were shown from three or more independent experiments. Bar, 50 μm.

Since the optimal pH_*e*_ value for MMP-9 induction was about pH 6.8, further experiments compared the effects of neutral (pH 7.4) and acidic (pH 6.8) pH_*e*_. At neutral pH_*e*_, LLCm1 cells had a cobble-stone appearance. When cultured at acidic pH_*e*_, however, their morphology became fibroblastic and of similar shape as LLCm4 cells (Figure 4). In contrast to LLCm1 cells, change in pH_*e*_ did not induce morphological changes in LLCm4 cells. These morphological observations suggested that acidic pH_*e*_ may induce EMT, however, the interesting lack of effect in the already mesenchymal cells should be mentioned.

### Acidic pH_*e*_ induces EMT-related gene expression

**Figure 5:**
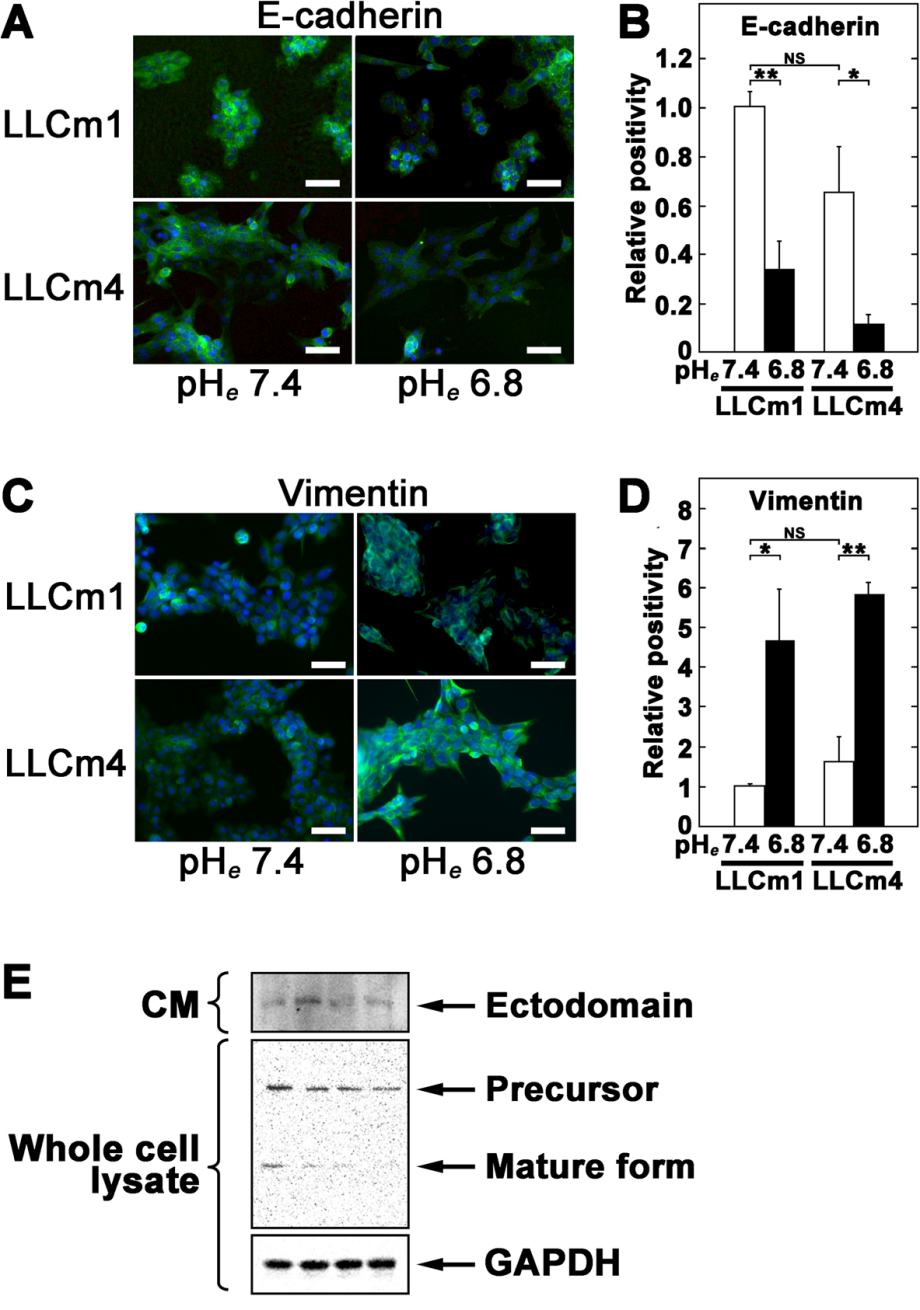
**Acidic pH**_***e***_ **induces vimentin but reduces E-cadherin expression.** Cultures were pretreated overnight with serum-free DMEM/12 at pH 7.4 and treated for 24 h with acidic serum-free medium at pH 6.8 and 7.4. The cells were subsequently fixed and incubated with antibodies to vimentin **(A & B)** and E-cadherin **(C & D)**. FITC-labeled signals were detected by fluorescence microscopy. **(A & C)** Representative results were shown from three or more independent experiments. Immuno-positivity was caliculated and their positivity was shown as relative values compared to LLCm1 cells at pH_*e*_ 7.4. Bar, 50 μm. *P < 0.05; **P < 0.01; NS, not significant. **E**. Western blot analysis for E-cadherin. After incubation of cells at pH 6.8, as described above, cells were lysed and analyzed by Western blotting. CM was concentrated and analyzed to detect shedded E-cadherin level in the CM.

**Figure 6:**
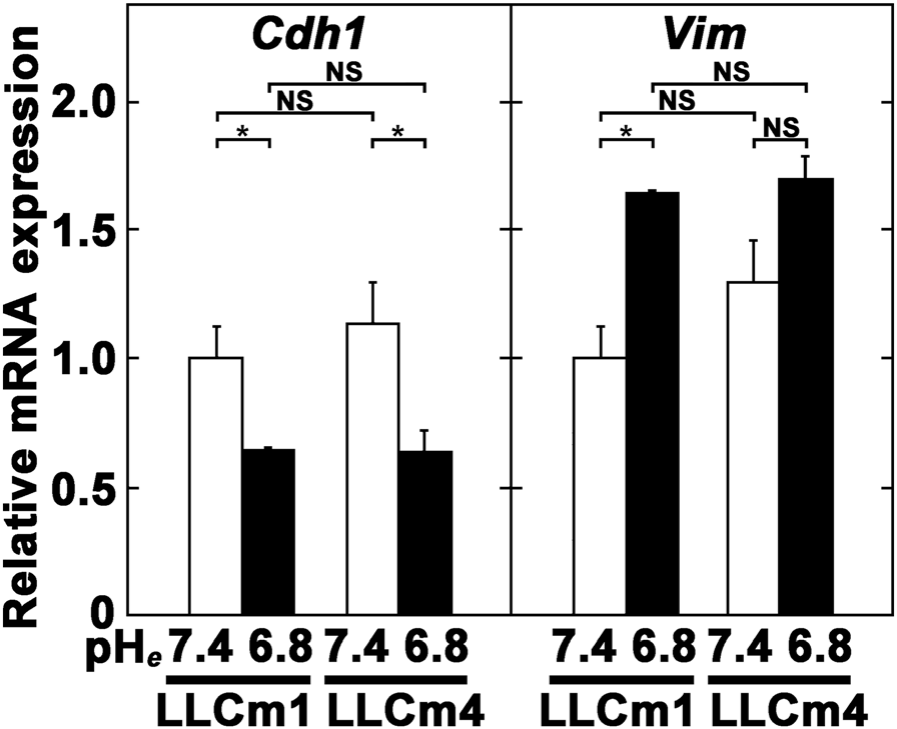
**Acidic pH**_***e***_ **induces** ***Vim*** **(vimentin) but reduces** ***Cdh1*** **(E-cadherin) mRNA expression.** Sub-confluent cultures were pretreated with serum-free medium at pH 7.4 and incubated in serum-free medium at pH 6.8 and 7.4. After 24 h, total RNA was extracted, reverse-transcribed, and amplified by qPCR with specific primer pairs. Data are shown as relative expression compared with their levels in LLCm1 cells cultured at pH 7.4. Representative results were shown from three independent experiments. *P < 0.05; NS, not significant.

To determine whether acidic pH_*e*_ induces EMT, we analyzed the expression of E-cadherin and vimentin using immunocytochemistry. In LLCm1 cells, E-cadherin and vimentin were significantly down- and up-regulated, respectively, by acidic pH_*e*_, findings typical of EMT (Figure 5). Interestingly, increase in ectodomain of E-cadherin was observed especially in LLCm1 cells (Figure 5E), suggesting that E-cadherin shedding was occurred by acidic pH_*e*_. Expression of *Cdh1* (E-cadherin) mRNA in both LLCm1 and LLCm4 cells was significantly decreased by incubation at acidic pH_*e*_ (Figure 6). Moreover, acidic pH_*e*_ increased the expression of *Vim* (vimentin) mRNA in both variants, but the difference was significant only in LLCm1 cells. Because LLCm4 is highly metastatic, vimentin expression may have been increased by other stimulatory systems, resulting in acidic pH_*e*_ having a weak effect on vimentin production by these cells.

**Figure 7:**
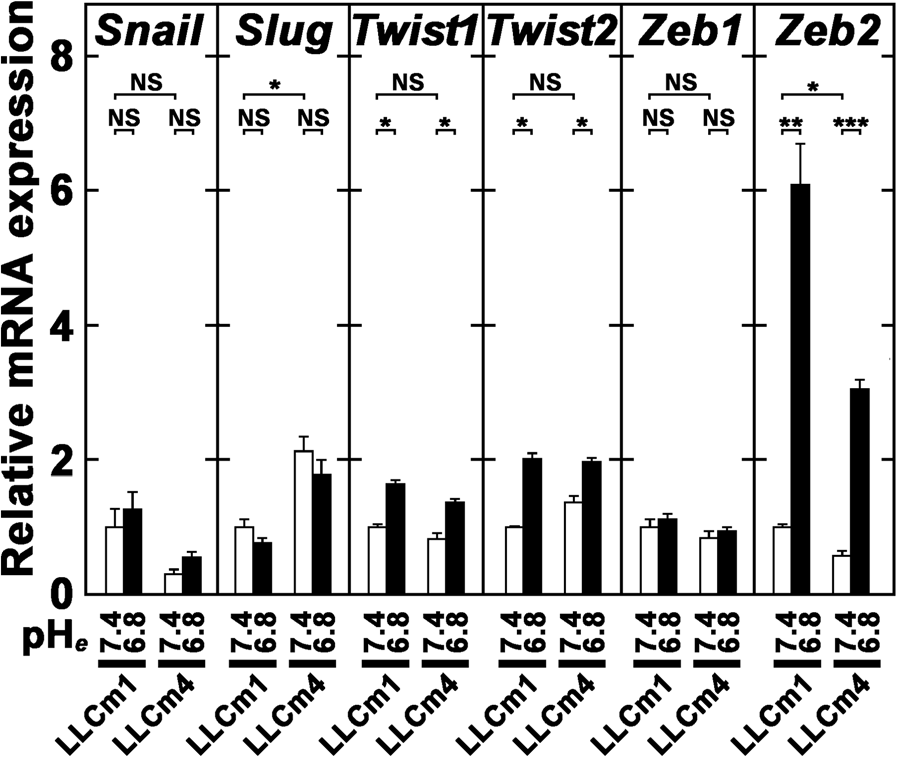
**Acidic pH**_***e***_ **induces** ***Twist1*** **,** ***Twist2 and Zeb2*** **but not** ***Snail*** **,** ***Slug, and Zeb1*** **mRNA expression.** Sub-confluent cells were washed twice with PBS(−), pretreated with serum-free medium at pH 7.4, and incubated in serum-free medium at the indicated pH. After 24 h, total RNA was extracted, reverse-transcribed, and amplified by qPCR with specific primer sets. Data are shown as relative expression compared with their levels in LLCm1 cells cultured at pH 7.4. Representative results were shown from three or more independent experiments. *P < 0.05; ***P < 0.001; NS, not significant.

Since several repressors of E-cadherin expression have been described, we tested the effects of acidic pH_*e*_ on the expression of these repressor(s) by RT-qPCR technology. Incubation at acidic pH_*e*_ induced steady state levels of *Twist1*, *Twist2*, and *Zeb2* mRNAs (Figure 7), with these levels inversely correlated with E-cadherin expression, suggesting that Twist1, Twist2, and Zeb2 were involved in acidic pH_*e*_ signaling that repressed E-cadherin expression. *Zeb2* mRNA level showed that highest degree of induction, suggesting that Zeb2 was primarily responsible for acidic pH_*e*_-induced EMT. In contrast, acidic pH_*e*_ had little effect on *Snail*, *Slug*, and *Zeb1* mRNA levels, suggesting that these molecules may be little involved in acidic pH_*e*_-induced EMT.

### Acidic pH_*e*_ induces cell migration and invasion

**Figure 8:**
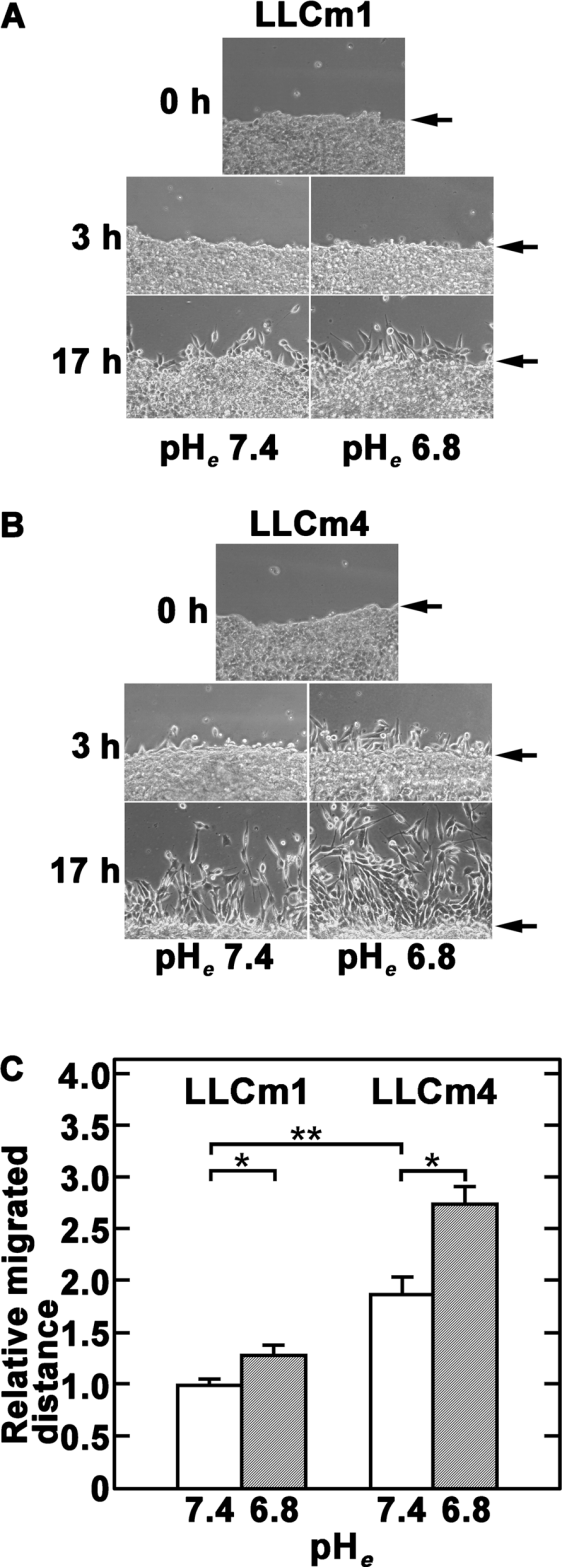
**Acidic pH**_***e***_ **induces migration activity.** Confluent cultures were scratched with micropipette tips and further cultured in 2% serum-containing medium adjusted to pH 7.4 or 6.8. Photographs were taken and cell migration was measured. **A**. Phase contrast micrograph. **B**. Migrated distance relative to LLCm1 cells at pH 7.4. Representative results were shown from three independent experiments (n = 8). *P < 0.05; **P < 0.01. **C**. Relative numbers of invasive cells compared with LLCm1 cells at pH 7.4.” between “**B**. Migrated distance relative to LLCm1 cells at pH 7.4.” and “Representative results were shown from three independent experiments (n = 8).

**Figure 9:**
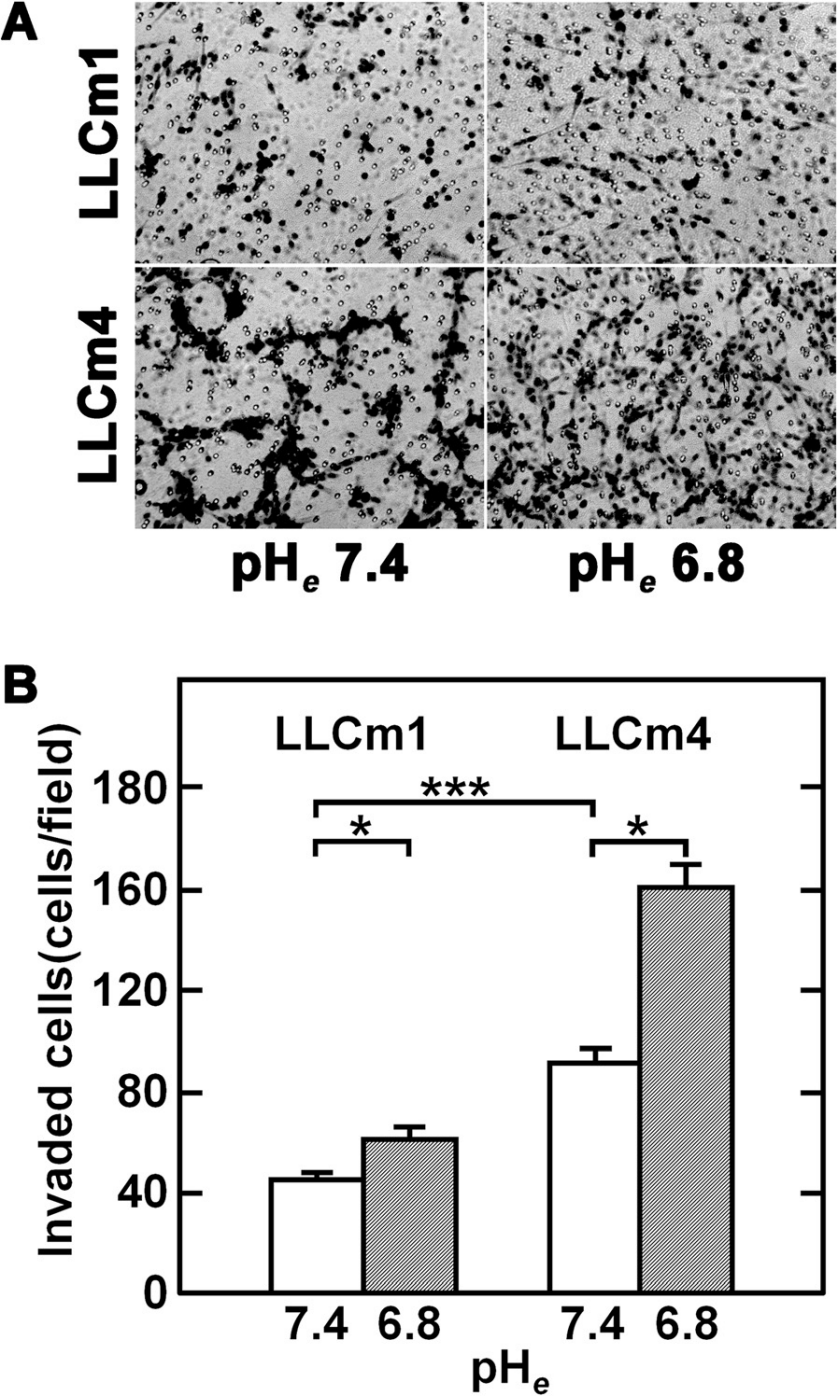
**Acidic pH**_***e***_ **induces invasive activity.** Confluent cultures were serum-starved and treated with serum-free medium at pH 7.4 or pH 6.8 for 18 h. The conditioned medium was collected and the cells were harvested by trypsinization. The cells were subsequently incubated with 10% FBS for 30 min to inhibit trypsin activity, washed twice with PBS(−) and resuspended in their own conditioned medium. The chemoattractant was 20% FBS. **A**. Phase contrast micrograph. **B**. Relative numbers of invasive cells compared with LLCm1 cells at pH 7.4. Representative results were shown from three independent experiments. *P < 0.05; ***P < 0.001.

Migration activity was determined using wound healing assays. Cells that formed a confluent sheet were scratched with a plastic tip, and the distance between the wound edge and the migration front were estimated. As expected, LLCm4 cells showed higher migration (Figure 8) and *in vitro* invasion (Figure 9) activities than LLCm1 cells. Both LLCm1 and LLCm4 cells migrated significantly further under acidic than neutral conditions. Furthermore, acidic pH_*e*_ stimulated the *in vitro* invasion activity of both cell lines.

## Discussion

Although reports using mouse [6] and human [7,9] melanoma models have shown that acidic pH_*e*_ induced EMT-like changes and increased invasive potential, the effects of acidic pH_*e*_ on EMT in carcinoma models were unclear. We therefore examined the effect of an acidic microenvironment on EMT in a carcinoma model using the LLC cell line. We found that acidic pH_*e*_ induces EMT in a carcinoma model, similar to findings in the melanoma model [6,7].

Typically, EMT includes the down-regulation of E-cadherin and the up-regulation of vimentin, N-cadherin, fibronectin, and MMP-9 expression [10,11]. The down-regulation of E-cadherin is driven by specific repressors, including Snail, Slug, Twist1, Twist2, Zeb1, and Zeb2 [1]. TGF-β induces EMT by increasing in *Zeb1*, *Snail*, *Slug*, and *Twist1* mRNA expression through Smad2 and Erk1/2 signaling [12]. In contrast, we found that acidic pH_*e*_-induced EMT was accompanied by up-regulation of expression of *Twist1*, *Twist2*, and *Zeb2* mRNAs, but not by changes in *Snail*, *Slug*, and *Zeb1* mRNA levels. In contrast, we found that TGF-β induces EMT along with MMP-9 expression [11], findings observed in acidic pH_*e*_. Therefore, aside from MMP-9 induction, EMT induction by acidic pH_*e*_ is via a somewhat different mechanism than TGF-β-triggered EMT.

NF-κB has been regarded as a key molecule for acidic pH_*e*_ signaling. NF-κB not only induces the expression of *Snail*, *Twist1*, *Slug*, and *Zeb2* mRNAs [13] but also blocks ubiquitination followed by the stabilization of Snail [14]. In some cases, NF-κB-induced EMT was accompanied by MMP-9 expression [15,16]. Metastatic cells of mesenchymal origin secrete abundant amounts of MMP-9 [17]. In addition, as we reported previously, acidic pH_*e*_ induces MMP-9 expression in mouse melanoma cells through NF-κB activation with change to a fibroblastic morphology [6,18,19]. These observations suggested that induction of MMP-9 expression through NF-κB was deeply associated with EMT.

TGF-β was shown to induce the expression of *MMP9* mRNA but decrease the expression of *MMP2* mRNA by human oral squamous cell carcinoma cell lines [20]. We have shown that acidic pH_*e*_ induces abundant MMP-9 production by the highly metastatic B16 variants, B16-F10 and B16-BL6 [6]. Although MMP-2 was expressed in parental B16 cell, little was expressed in the low and high metastatic variants, B16-F1, B16-F10 and B16-BL6. Using an LLC model, we observed findings similar to those in the B16 melanoma lines. Reduction of MMP-2 activation was reported due to increased intracellular Ca^2+^ [21]. In contrast, we previously showed that elevation of intracellular Ca^2+^ induced MMP-9 expression [19]. Although acidic pH_*e*_ induces a Ca^2+^ influx, its mechanism of expression of MMP-2 is still unclear.

EMT includes the suppression of the epithelial marker E-cadherin. Immunocytochemical staining showed that incubation in acidic pH_*e*_ markedly reduced E-cadherin expression, but had little effect on the expression of *Cdh1* (E-cadherin) mRNA. Acidic pH_*e*_ did not affect the half life of *Cdh1* mRNA level (data not shown). Interestingly, E-cadherin was increased in CM, especially in LLCm2 cells, by acidic pH_*e*_. Because MMP-9 catalyzes E-cadherin ectodomain shedding during EMT [22,23], acidic pH_*e*_-induced MMP-9 production may not only promote extracellular matrix degradation but also E-cadherin shedding to induce EMT.

Signal cross talk is also important in EMT. For example, in an oral squamous cell carcinoma model, the combination of TGF-β1 and EGF, but not either alone, induced cell scattering activity [24]. Similarly, TGF-β sensitizes cells to basic FGF/FGF-2 stimulation to induce EMT [25]. Thus, intracellular signaling pathways seem to be complex. Acidic pH_*e*_ can arise through the production of lactate and/or the generation of excess amounts of CO_2_ through the pentose-phosphate pathway [26]. Because hypoxia induces EMT [27], acidic pH_*e*_-induced EMT may be modulated by hypoxia.

Acidic pH_*e*_ induces the expression of many genes, including MMP-9 [6,28], VEGF-A [29-31], VEGF-C [32], interleukin-8 [33-35], the inducible isoform of nitric oxide synthase (iNOS) [36], platelet-derived endothelial cell growth factor (PDGF)/thymidine phosphorylase [37], and acidic sphyngomyelinase [19], as well as Twist1, Twist2, Zeb2, vimentin, MMP-3, and MMP-13, which were shown in this study. In addition, acidic pH_*e*_ can affect cell migration, stress fiber formation, invasion and metastasis [4,9]. Thus, acidic pH_*e*_ acts as a microenvironmental factor that promotes malignant phenotypes, such as metastatic ability.

LLC is thought to originate from a cancer derived from granular pneumocytes and to be equivalent to a human alveolar cell carcinoma (squamous cell carcinoma) [38]. Therefore, LLC is regarded as a mouse model of non-small cell lung cancer [39,40]. Acidic pH_*e*_-induced EMT may also be involved in the origins of other types of squamous cell carcinoma such as head and neck and esophageal cancers.

## Conclusion

These findings suggest that acidic pH_*e*_ constitutes an important microenvironment that induces EMT in some types of carcinoma.

## Methods

### Reagents

Dulbecco’s modified Eagle medium (DMEM), Ham’s F12 medium, and High Capacity RNA-to-cDNA kit were purchased from Life Technologies (Grand Island, NY, USA). Realtime PCR master mix was from Takara (Tokyo, Japan). Anti-vimentin polyclonal antibodies was from ImmunoResearch Laboratories (Grove, PA, USA). Anti-E-cadherin monoclonal antibody was from Santa Cruz (G-10, Santa Cruz, CA, USA) Avidin-conjugated fluorescein isothiocyanate (FITC) was from Vector Laboratories (Burlingame, CA, USA), and N102 blocking reagent was from NOF Corporation (Tokyo, Japan). Fetal bovine serum (FBS) was from Hyclone (South Logan, UT, USA). DC protein assay kits (based on the Lowry method) were from Bio-Rad Laboratories (Hercules, CA, USA), and Isogen RNA extraction kits were from Nippon Gene (Tokyo, Japan). Transwell chambers with 8 μm pores were from BD Bioscience (Franklin Lakes, NJ, USA). Matrigel® was from Corning (Tewksbury, MA, USA).

### Cells and cell culture

The LLC cell line, a squamous cell carcinoma cell line derived from granular pneumocytes and equivalent to human alveolar cell carcinoma [38], was the kind gift of Dr. Ryu-Ichro Hata (Kanagawa Dental University, Yokosuka, Japan). LLC cells were cultured in DMEM/F12 (a 1:1 mixture of DMEM and Ham’s F12 media, adjusted to pH 7.4 with 15 mM HEPES, 4 mM phosphorus, 1 mg/ml NaHCO_3_), supplemented with 10% FBS at 37°C in a humidified atmosphere of 5% CO_2_ and 95% air. The cells were passaged with 0.05% trypsin/0.02% EDTA. The pH of the medium was adjusted with NaOH or HCl and measured after incubation in the CO_2_ incubator for 3 h.

### Experimental metastasis and *in vivo* selection of metastatic variants

Cells (3 × 10^5^ cells) were resuspended in 200 μl Ca^2+^- and Mg^2+^-free Dulbecco’s phosphate-buffered saline (PBS(−)) and injected into the tail vein of a C57BL/6 mouse (Clea Japan, Tokyo, Japan). Three weeks later, the mice were sacrificed under anesthesia with an excess dose of pentobarbital and the metastasized foci in the lungs were counted.

Variants with different metastatic potential were established as described [41]. Briefly, LLC parental cells were injected into the tail vein of a syngeneic C57BL/6 mouse. Three weeks later, the mice were sacrificed, and metastasized foci were taken from the lungs, treated with trypsin/EDTA and cultured in DMEM/F12 + 10% FBS. These cells, referred to as LLCm1 cells, were again injected into mouse tail veins and metastasized foci in the lungs were cultured. The cells obtained after 4 cycles were the high metastatic variant, referred to as LLCm4 cells.

All animal protocols were approved by the Animal Use Committee of Ohu University.

### Preparation of conditioned medium for zymography

Conditioned media were prepared essentially as described [6], with modifications. Briefly, serum-free conditioned media were taken from cultured cells, and proteins were precipitated by addition of 2.5 volumes of acetone. The samples were reconstituted in PBS(−) or 0.5 mM Tris–HCl (pH 6.8) supplemented with 1% SDS and 0.2% glycerol.

### Zymography

Zymography on gelatin-containing sodium dodecyl sulfate (SDS)-7.5% polyacrylamide gels was performed as described [19]. Briefly, concentrated samples were electrophoresed in 0.1% gelatin-containing 7.5% polyacrylamide gels. The gels were washed with 2.5% Triton X-100 at room temperature with gentle shaking for 1 h and incubated for 20 h in reaction buffer (50 mM Tris–HCl (pH 7.5), 10 mM CaCl_2_) at 37°C. Gelatinolytic activity was visualized by Coomassie Brilliant Blue R250 staining. Activity was normalized relative to cell number or protein concentration, with the latter determined by the Lowry method using bovine serum albumin as the standard.

### RT-qPCR

Total RNA was extracted by Isogen and transcribed into cDNA using reverse transcriptase. Quantitative PCR was performed using specific primer sets (Table 1) according to the manufacturer’s protocol.

**Table 1:** **Primer sets for RT-qPCR analysis**

**Gene**		**Sequences**	**Product size (bp)**	**Accession number** ^*^
*β-actin*	Forward:	5′-CATCCGTAAAGACCTCTATGCCAAC-3′	186	NM_007393
	Reverse:	5′-ATGGAGCCACCGATCCACA-3′		
*Mmp2*	Forward:	5′-AGGCAGTAGAGTAAGGGGATCG-3′	279	NM_008610.2
	Reverse:	5′-TAGAAAGTGTTCAGGTATTGCACTG-3′		
*Mmp3*	Forward:	5′-TGAAGCATTTGGGTTTCTCTACT-3′	134	NM_010809
	Reverse:	5′-GATGCCTTCCTTGGATCTCTTT-3′		
*Mmp9*	Forward:	5′-GCCCTGGAACTCACACGACA-3′	85	NM_013599
	Reverse:	5′-TTGGAAACTCACACGCCAGAAG-3′		
*Mmp13*	Forward:	5′-TCCCTGGAATTGGCAACAAAG-3′	120	NM_008607.2
	Reverse:	5′-GCATGACTCTCACAATGCGATTAC-3′		
*Mmp14*	Forward:	5′-TCTTCAAGGAGCGATGGTTCT-3′	182	NM_008608.3
	Reverse:	5′-CAGGGAGGCTTCGTCAAACA-3′		
*Vim*	Forward:	5′-GGACGTTTCCAAGCCTGACCTC-3′	198	NM_011701
	Reverse:	5′-CCGGTACTCGTTTGACTCCTGC-3′		
*Cdh1*	Forward:	5′- ATTGCAAGTTCCTGCCATCCTC -3′	145	NM_009864
	Reverse:	5′-CACATTGTCCCGGGTATCATCA-3′		
*Cdh3*	Forward:	5′-TCGTGAGGACGAGCAGTTTG-3′	130	NM_007665
	Reverse:	5′-GCCATGGTCGTTGATGTCAG-3′		
*Snail*	Forward:	5′-AGGACGCGTGTGTGGAGTTC-3′	235	NM_011427
	Reverse:	5′-TGGGAGCTTTTGCCACTGTC-3′		
*Slug*	Forward:	5′-CATTCGAACCCACACATTGCC-3′	112	NM_011415
	Reverse:	5′-AGAGAAAGGCTTTTCCCCAGTG-3′		
*Twist1*	Forward:	5′-GCCGGAGACCTAGATGTCATTG-3′	149	NM_011658
	Reverse:	5′-ACGCCCTGATTCTTGTGAATTTG-3′		
*Twist2*	Forward:	5′-GCAAGCCAGGACCCACC-3′	100	NM_007855
	Reverse:	5′-GTCATGAGGAGCCACAAGGT-3′		
*Zeb1*	Forward:	5′-GCTGGCAAGACAACGTGAAAG-3′	116	NM_011546
	Reverse:	5′-AGGATAAATGACGGCGGTGT-3′		
*Zeb2*	Forward:	5′-AGACTTCACAGATCGAGCCT-3′	146	NM_015753
	Reverse:	5′-CCTCCTGGGATTGGCTTGTT-3′		

^*^Accession number of the National Center for Biotechnology Information (NCBI).

### Immunocytochemistry

Cells were fixed in 4% paraformaldehyde and blocked with 20% N102 blocking reagent in Tris-buffered saline (20 mM Tris, pH 7.5, 150 mM NaCl, supplemented with 0.05% Tween 20 (TBS-T). The cells were incubated overnight at 4°C with primary antibody, washed extensively, incubated with biotin-conjugated secondary antibody, washed, and treated with avidin-conjugated fluorescein isothiocyanate (FITC). The cells were viewed by fluorescence microscopy (EVOS® FLoid® Cell Imaging Station, Life Technologies, Carlsbad, CA, USA).

### Wound healing (scratch) assay

Wound healing assays were performed as described, with slight modifications [42]. Briefly, confluent cultures were serum-starved for 24 h and scratched off with a micropipette tip. The cells were cultured in medium containing 0.2% FBS at pH 7.4 or pH 6.8. After indicated time period, photographs were taking and estimated wound distance.

### *In vitro* invasion assay

*In vitro* invasive activity was determined using Matrigel®-coated filter mounted transwell chambers (Corning, Tewksbury, MA, USA) as described [43]. Briefly, cells were serum-starved overnight at pH 7.4 and then further maintained in serum-free media at pH 7.4 or 6.8 for 18 h. The cells were harvested with 0.05% trypsin/0.02% EDTA and incubated at 37°C for 30 min in medium containing 10% FBS to inhibit trypsin activity. The cells were washed twice with PBS(−), re-suspended in serum-free medium at pH 7.4 or 6.8, and inoculated at density of 5 × 10^5^ cells/100 μl/chamber onto a Matrigel® (37.9 μg/cm^2^)-coated filter in the insert, which had been mounted onto a well of a 24 well plate, with each well filled with 600 μl 10% FBS-containing medium adjusted to each pH. After incubation for 18 h, non-invasive cells were scraped off with a cotton swab and invasive cells were fixed in 100% methanol, stained with hematoxylin, and counted under light microscope (×200).

### Statistical analysis

Between group differences were compared using Student’s *t*-tests or ANOVA. Statistical significance was defined as a p value less than 0.05.

## Abbreviations

EMT: Epithelial mesenchymal transition
FGF: Basic fibroblast growth factor
TGF-β: Transforming growth factor-β
pH_*e*_: Extracellular pH
MMP-9: Matrix metalloproteinase-9
LLC: Lewis lung carcinoma
RT-qPCR: Reverse transcription-quantitative polymerase chain reaction
DMEM: Dulbecco’s modified Eagle medium
FBS: Fetal bovine serum
PBS(−): Ca^2+^- and Mg^2+^-free Dulbecco’s phosphate-buffered saline
